# The role of Bells in the continuous accretion between the CM and CR chondrite reservoirs

**DOI:** 10.1111/maps.13459

**Published:** 2020-03-09

**Authors:** Elishevah van Kooten, Larissa Cavalcante, Daniel Wielandt, Martin Bizzarro

**Affiliations:** ^1^ Institut de Physique du Globe de Paris Université de Paris CNRS UMR 7154 1 rue Jussieu 75238 Paris France; ^2^ Institute of Chemistry University of São Paulo 03178 São Paulo Brazil; ^3^ Centre for Star and Planet Formation and Natural History Museum of Denmark University of Copenhagen DK‐1350 Copenhagen Denmark

## Abstract

CM meteorites are dominant members of carbonaceous chondrites (CCs), which evidently accreted in a region separated from the terrestrial planets. These chondrites are key in determining the accretion regions of solar system materials, since in Mg and Cr isotope space, they intersect between what are identified as inner and outer solar system reservoirs. In this model, the outer reservoir is represented by metal‐rich carbonaceous chondrites (MRCCs), including CR chondrites. An important question remains whether the barrier between MRCCs and CCs was a temporal or spatial one. CM chondrites and chondrules are used here to identify the nature of the barrier as well as the timescale of chondrite parent body accretion. We find based on high precision Mg and Cr isotope data of seven CM chondrites and 12 chondrules, that accretion in the CM chondrite reservoir was continuous lasting *<*3 Myr and showing late accretion of MRCC‐like material reflected by the anomalous CM chondrite Bells. We further argue that although MRCCs likely accreted later than CM chondrites, CR chondrules must have initially formed from a reservoir spatially separated from CM chondrules. Finally, we hypothesize on the nature of the spatial barrier separating these reservoirs.

## Introduction

Unravelling the accretion regions of planetary building blocks such as chondrites and their components is critical for a full understanding of the evolution and dynamics of the solar system. Recent progress in determining the accretion regions of chondrites has come from isotope studies, where nucleosynthetic isotope variability is commonly used to probe the genetic relationship between various meteorites and their parent bodies. For example, Trinquier et al. (2009) observed a clear divide in Ti versus Cr isotope space between noncarbonaceous (NC) and carbonaceous chondrites (CC), indicating a taxonomic difference between the two groups. This bimodal distribution is also observed for a number of different nuclides, including oxygen (Qin et al. 2010; Warren 2011). Originally suggested by Trinquier et al. (2009) but subsequently reinforced by a Mg and Cr isotope study of bulk chondrites (Larsen et al. 2011), the bimodality is thought to reflect thermal processing of pristine dust that results in the inmixing of various presolar components and, hence, nucleosynthetic variability. In detail, a positive correlation between ^26^Mg* (the decay product of ^26^Al) and ^54^Cr is observed between solar system reservoirs with solar or near‐solar Al/Mg ratios. The solar system's ^26^Mg*‐^54^Cr correlation line is attributed to preferential destruction of a ^26^Al and ^54^Cr‐rich labile dust component derived from supernovae over a relatively ^26^Al‐ and ^54^Cr‐poor dust component represented by more robust, galactically inherited dust, before the accretion of this dust into chondrite parent bodies. In this model, thermal processing is most extensive in the innermost disk regions characterized by high temperatures, resulting in the coexistence of two complementary reservoirs with contrasting abundances of ^26^Al and ^54^Cr. The first solar system solids, calcium‐aluminum inclusions (CAIs), are proposed to have condensed from the gaseous reservoir enriched in ^26^Al and ^54^Cr relative to bodies that accreted from the residual dust (Trinquier et al. 2009). With larger orbital distance in the accretion regions of CCs, thermal processing becomes less pronounced resulting in more ^26^Mg*‐ and ^54^Cr‐rich materials. Although this model has not reached general consensus, thermal processing of dust in the protoplanetary disk has subsequently been invoked to explain other isotope heterogeneities in bulk chondrites including nucleosynthetic variations from Sr, Ca, Mo, and W (Burkhardt et al. 2011; Paton et al. 2013; Schiller et al. 2015b, 2018; Poole et al. 2017).

The metal‐rich carbonaceous chondrites (MRCCs) that include CR (Renazzo‐type), CB (Bencubbin‐type), and CH chondrites do not fall on the solar system's ^26^Mg*‐^54^Cr correlation line (Van Kooten et al. 2016). These chondrites are thought to have accreted late in the evolution of the protoplanetary disk (Krot et al. 2005; Bollard et al. 2015; Schrader et al. 2017; Budde et al. 2018) and it is generally accepted that CH and CB chondrites formed from an impact melt plume around 5 Myr after CAI formation (Krot et al. 2005; Bollard et al. 2015). The MRCCs are characterized by a high abundance of metal and extreme enrichments in ^15^N (Krot et al. 2002), the latter being commonly observed in N‐bearing ices from the interstellar medium (Hily‐Blant et al. 2013). These data suggest that MRCCs accreted in a reservoir isolated from that of other thermally processed solar system materials that are thought to have formed in a reservoir located sunward of the MRCCs. In detail, these chondrites are believed to have accreted beyond the orbits of the gas giants and incorporated significant amounts of thermally unprocessed, primordial molecular cloud material (Van Kooten et al. 2016). This interpretation is consistent with structural and N isotope observations of organic matter in chondritic lithic clasts from the Isheyevo CH/CB_*b*_ meteorite, which suggests that these clasts accreted in regions where NH_3_ and HCN ices were stable (e.g., *>*5 AU; Van Kooten et al. 2017a). However, an outstanding issue is whether chondritic reservoirs were spatially or temporally separated from each other.

It has been proposed that the distinct isotopic flavor of the NCs and CCs is the result of a spatial barrier in the protoplanetary disk raised by the rapid accretion of Jupiter (Budde et al. 2016; Van Kooten et al. 2016; Kruijer et al. 2017). Similarly, Mg and Cr isotope variations between CCs and MRCCs may reflect spatial isolation by barriers including the other gas giants. However, Mg and Cr isotope analyses of individual chondrules from CV (Vigarano‐type) and CR chondrites suggest that some CV chondrules incorporated material from the MRCC reservoir (Olsen et al. 2016), indicating that (1) the proposed spatial barrier was to some degree permeable and allowed for inward mass transport or (2) the isotope distinction predominantly reflects time and not space. To investigate the nature of the difference between MRCCs and CCs, we focus on CM (Mighei‐type) chondrites, which in ^54^Cr versus ^26^Mg* space overlap between the two outer solar system reservoirs (Van Kooten et al. 2016). Hence, CM chondrites are essentially at the frontier of either a spatial or temporal transition in the protoplanetary disk.

Previous Mg and Cr isotope measurements of CM chondrites include a single bulk analysis of Jbilet Winselwan, a moderately altered breccia. Here, we have increased the number of bulk CM chondrites to seven, with a range of petrological type from CM2.1 to CM2.7, and report on the Mg and Cr isotope compositions of 12 individual chondrules from Jbilet Winselwan. We discuss the timescale of accretion for the CM chondrite parent body, the origin of chondrules, and finally propose a hypothesis on the nature of the barrier between MRCCs and CCs.

## Materials and Methods

We have selected seven bulk CM chondrites and 12 porphyritic olivine chondrules from the CM chondrite Jbilet Winselwan. Among our selection of bulk chondrites are the well‐documented CM chondrites Murchison (CM2.5, Rubin et al. 2007), Murray (CM2.4/2.5, Rubin et al. 2007), Cold Bokkeveld (CM2.2, Rubin et al. 2007), and the prototypical Mighei (CM2.3, Rubin et al. 2007) as well as the relatively unaltered Maribo (CM2.7, Haack et al. 2012; Van Kooten et al. 2018), and the anomalous Bells (CM2.1/2.2, Van Kooten et al. 2018). Murchison, Mighei, Murray, Bells (the un‐weathered fall), and Cold Bokkeveld are part of the permanent collections of the Natural History Museum of Denmark and were acquired in 1973, 1906, 1991, 1995, and 1862, respectively. These meteorites were curated according to standards at the time of their acquisition, which entails that they were stored in non‐hermetic vials. In contrast, Maribo was collected after it fell in Denmark in 2009 and was stored according to a more modern standard, which includes hermetically sealed containers. Jbilet Winselwan was acquired from a meteorite dealer in 2014 and is a part of a research collection at the Natural History Museum of Denmark and has been stored in non‐hermetic vial.

From the chondrites, approximately 150 mg of material was taken to ensure a good representation of the bulk composition. The chondrites were subsequently crushed in an agate mortar with ethanol and transferred to clean Savilex beakers. From the chondrules, the entire chondrule core (e.g., without dust rim) was sampled using a computer‐assisted NewWave micro‐drill with tungsten carbide drill bits at the Centre for Star and Planet Formation (Copenhagen). Powders from the drilled samples were transferred with double distilled MQ water to clean Savillex beakers. Care was taken not to drill close to the chondrule boundaries to avoid contamination from surrounding matrix. The individual drill spots were examined under a microscope for possible contamination and if contamination was suspected, the potentially affected samples were discarded.

The samples were dissolved on a hotplate for 2 days at 150 ^°^C in concentrated HNO_3_/HF acid and subsequently in Parr bombs for 1 day at 210 ^°^C. The solutions were then dried down and treated with aqua regia for another day, resulting in a complete digestion of the sample powders. To determine the ^27^Al/^24^Mg ratios, a 5% aliquot was taken from each sample solution before starting the purification process. These aliquots were analyzed with an accuracy of 2% on the ThermoFisher X‐series quadrupole ionization coupled plasma mass spectrometer (ICPMS) at the Centre for Star and Planet Formation (Copenhagen). The subsequent purification of Mg and Cr using column chromatography was done by methods described in Bizzarro et al. (2011) and Van Kooten et al. (2016).

The isotopic composition of the purified Mg was determined with a standard sample bracketing technique using the Neptune Plus Multi Collector (MC) ICPMS at the Natural History Museum of Denmark based on protocols described in Bizzarro et al. (2011) and Van Kooten et al. (2016). Samples were typically analyzed with a signal intensity of 20–45 V on mass ^24^Mg for the smaller chondrules (typically *<*50 μg of Mg) and 150 V for larger bulk samples. Each sample was systematically analyzed 10 times. Mg‐isotope data are reported in the μ‐notation as deviations from the DTS‐2b standard (μ^25^Mg_*DSM*−3_ = −122 ± 17 ppm [2SD, Bizzarro et al. 2011]) according to the following formula:

(1) $$\mu^{x}Mg\;\left(\mathrm{ppm}\right)=\left[\frac{xMg/24Mg_{\mathrm{sample}}}{xMg/24Mg_{DTS-2b}}-1\right]\times 10^{6}$$

where x represents mass 25 or 26. The mass‐independent component of ^26^Mg (μ^26^Mg*) is reported in the same fashion, but represents deviations from the internally normalized ^26^Mg/^24^Mg of the sample from the reference standard, normalized to ^25^Mg/^24^Mg = 0.126896 (Bizzarro et al. 2011) using the exponential mass fractionation law. All Mg data reduction was conducted off‐line using the Iolite data reduction package, which runs within Igor Pro (Paton et al. 2012). Within this software, changes in mass bias with time were interpolated using a smoothed cubic spline. For each analysis, the mean and standard error of the measured ratios were calculated using a 2SD threshold to reject outliers. Individual analyses of a sample were combined to produce an average weighted by the propagated uncertainties of individual analyses and reported final uncertainties are the 2SE of the mean. The external reproducibility of our measurements using this method is 20 and 2.5 ppm for the μ^25^Mg and μ^26^Mg*, respectively Bizzarro et al. (2011).

The Cr‐isotope composition of all samples was measured by thermal ionization mass spectrometry (Triton TIMS) at the Centre for Star and Planet Formation. We used the small chemistry column purification techniques described by Schiller et al. (2014) and adapted by Van Kooten et al. (2017b).

Chromium isotope analyses were conducted using a hybrid method of total evaporation and standard sample bracketing fully described in Van Kooten et al. (2016). Samples were typically measured 16 times distributed over multiple sessions. The SRM‐3112a standard was measured concurrently over all sessions. Cr‐isotope data are presented in the μ‐notation relative to this standard:

(2) $$\mu^{x}Cr\;\left(\mathrm{ppm}\right)=\left[\frac{xCr/52Cr_{\mathrm{sample}}}{xCr/52Cr_{SRM-3112a}}-1\right]\times 10^{6}$$

where x represents mass 53 and 54. Individual analyses of a sample were combined to produce an average weighted by the propagated uncertainties of individual analyses and reported final uncertainties are the 2SE of the mean using a 2SD outlier rejection scheme. This setup resulted in an external reproducibility of 6 ppm (2SD) and 12 ppm on mass‐bias corrected μ^53^Cr and μ^54^Cr values, respectively, when using a run number of 16 filaments (Van Kooten et al. 2016).

## Results

### Bulk CM Chondrites

**Figure 1:**
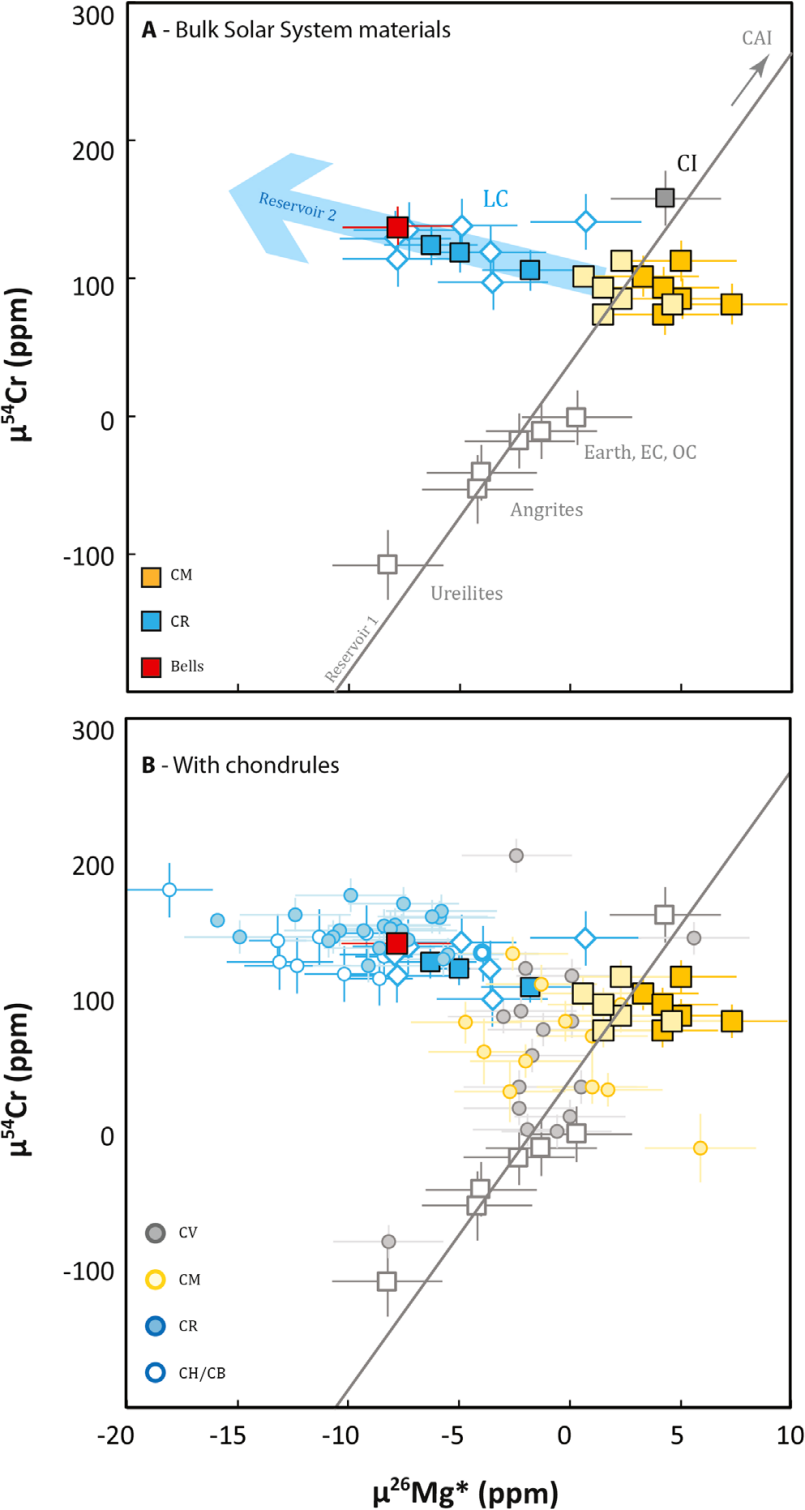
A) Mg and Cr isotope data for bulk solar system materials modified after Van Kooten et al. (2016), where objects lying on the positive correlation between μ^26^Mg* and μ^54^Cr (gray line) are defined as “reservoir 1” (e.g., non‐carbonaceous, Earth, carbonaceous chondrites [CMs in yellow, with lighter yellow depicting CMs corrected for ingrowth of μ^26^Mg*, see text for further information], calcium‐aluminum inclusions). Reservoir 2 is represented by objects lying off the positive correlation line, namely metal‐rich carbonaceous chondrites (blue) and Bells (red). Bulk materials are represented by squares, Isheyevo lithic clasts by diamonds, and chondrules by spheres. Errors are assigned according to the external reproducibility of the measurements (e.g., 2.5 ppm for μ^26^Mg* and 12 ppm for μ^54^Cr), unless the error is larger. B) Data from individual CV, CR Van Kooten et al. (2016); Olsen et al. (2016) and CM (this work) chondrules plotted over bulk chondrite data from Fig. 1A. (Color figure can be viewed at http://www.wileyonlinelibrary.com.)

**Figure 2:**
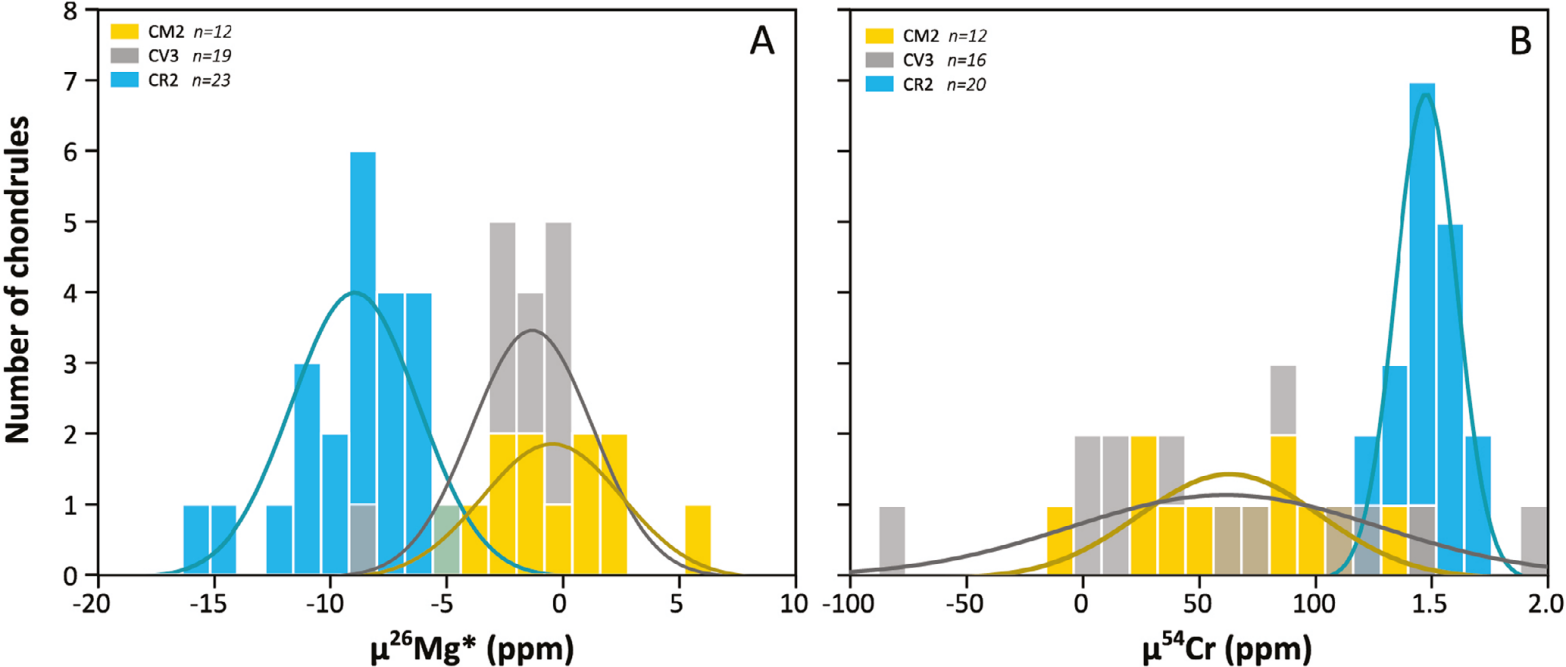
Histograms for chondrule populations from CR (blue), CV (gray), and CM (yellow) chondrites depicting distributions of (A) μ^26^Mg* and (B) μ^54^Cr values. The curves in the plots show Gaussian fits to the data from Past3.14 software. (Color figure can be viewed at http://www.wileyonlinelibrary.com.)

We report on the Mg and Cr isotope compositions and ^27^Al/^24^Mg ratios of seven bulk CM chondrites (Table 1), including Bells and Maribo, as well as more typical CM chondrites Murchison, Mighei, Murray, and Cold Bokkeveld. We show that, apart from Bells, CM chondrites have very consistent μ^26^Mg* values averaging at 4.6 ± 2.8 ppm (2SD). This average value is indistinguishable from CI chondrites (μ^26^Mg* = 4.5 ± 2.5 ppm; Larsen et al. 2011). However, the marginally different ^27^Al/^24^Mg ratios of these CM chondrites (0.111 ± 0.006, 2SD) relative to the solar value based on CI chondrites (0.09781 ± 0.00029; Paton et al. 2012) correspond to an additional 2.7 ppm of ingrowth assuming an initial ^26^Al/^27^Al ratio of 2.8 × 10^−5^ for the CI‐forming reservoir (Larsen et al. 2011). Correcting for this ingrowth brings the average CM μ^26^Mg* composition on the solar systems’ μ^26^Mg* – μ^54^Cr correlation (Fig. 1A), which is more consistent given its lower μ^54^Cr relative to CI chondrites. The μ^26^Mg* value for Bells is significantly lower (μ^26^Mg* = −7.8 ± 0.9 ppm) than the other CM and CI chondrites, despite having a similar ^27^Al/^24^Mg ratio (0.101, Fig. 2A). The μ^25^Mg value of Bells is within error of the average μ^25^Mg value of other CM chondrites (7 ± 40 ppm, 2SD).

**Table 1:** Mg and Cr isotope data for bulk CM and CR chondrites and CM chondrules from Jbilet Winselwan relative to the DTS‐2b and SRM979 standard, respectively. Errors are in 2SE, except for average compositions that are 2SD

Samples	^27^Al/^24^Mg	μ^25^Mg (ppm)	μ^26^Mg^a^ (ppm)	^55^Mn/^52^Cr	μ^54^Cr (ppm)	μ^53^Cr (ppm)
Standards						
BHVO‐2		−18 ± 2	1.2 ± 2.4		4 ± 8	1 ± 6
CM chondrites						
Jbilet (1)^a^	0.114	35 ± 7	2.8 ± 1.2	0.629	101 ± 12	19 ± 6
Jbilet (2)^a^	0.116	23 ± 6	3.8 ± 1.2			
Mighei	0.109	6 ± 30	4.2 ± 1.6	0.634	74 ± 10	18 ± 3
Cbok	0.110	12 ± 10	7.3 ± 1.0	0.645	81 ± 12	7 ± 3
Murray	0.111	−20 ± 9	5.0 ± 1.3	0.628	85 ± 10	18 ± 3
Maribo	0.110	10 ± 6	5.0 ± 2.1	0.647	113 ± 15	29 ± 4
Murchison	0.107	−18 ± 8	4.2 ± 1.4	0.669	93 ± 7	19 ± 4
Average	0.111	7 ± 40	4.6 ± 3	0.642	91 ± 28	18 ± 14
Bells	0.101	10.1 ± 7	−7.8 ± 0.9	0.623	137 ± 13	17 ± 4
CM chondrules (Jbilet)						
J1_3	0.096	−53 ± 18	2.3 ± 2.6		93 ± 20	2 ± 6
J1_21	0.057	−84 ± 13	−2.6 ± 2.0		130 ± 20	26 ± 6
S1_8	0.145	−27 ± 22	5.9 ± 2.0	0.162	−11 ± 40	−9 ± 6
S1_6	0.126	−94 ± 11	1 ± 2.4	0.356	70 ± 40	8 ± 13
S3_6	0.075	−18 ± 15	−2 ± 1.5	0.194	52 ± 7	9 ± 2
S3_20	0.051	192 ± 9	−3.9 ± 1.5	0.173	59 ± 38	6 ± 9
S1_25	0.076	−46 ± 13	−1.3 ± 2.5	0.203	108 ± 21	11 ± 5
S1_22	0.090	21 ± 9	−2.7 ± 2.4	0.174	30 ± 35	−7 ± 6
S2_24	0.052	−110 ± 11	−4.7 ± 2.1	0.215	80 ± 23	22 ± 4
S3_7	0.062	−64 ± 6	1 ± 1.1	0.194	33 ± 20	8 ± 2
S2_21	0.065	3 ± 3	1.7 ± 1.8	0.128	31 ± 20	2 ± 6
S2_25R	0.051	−108 ± 3	−0.2 ± 2.5	0.168	81 ± 24	8 ± 7
Average	0.079	−32 ± 165	−0.50 ± 6.0	0.197	63 ± 78	7 ± 20
CR chondrites^a^						
NWA 6043	0.096	13 ± 14	−6.3 ± 2.1	0.575	124 ± 10	7 ± 7
EET 92161	0.128	−88 ± 10	−5.0 ± 1.4	0.499	119 ± 12	21 ± 4
NWA 7837	0.137	−52 ± 6	−1.8 ± 2.2	0.386	106 ± 8	2 ± 4
Average	0.12	−42 ± 102	‐4.4 ± 5	0.487	116 ± 19	10 ± 20

a Data from Van Kooten et al. (2016).

Finally, the CM chondrites—except Bells—have μ^54^Cr compositions ranging between 74 ± 10 and 101 ± 12 ppm, with an average of 91 ± 11 (2SD) ppm. Bells’ μ^54^Cr signature is significantly higher than the other CM chondrites (μ^54^Cr = 137 ± 13 ppm), whereas the ^55^Mn/^52^Cr ratio of Bells (^55^Mn/^52^Cr = 0.623) and the corresponding μ^53^Cr value (17 ± 4 ppm) are indistinguishable from the other CM chondrites (average ^55^Mn/^52^Cr = 0.639 and μ^53^Cr = 20 ± 14 ppm, 2SD). Collectively, our data show that while the μ^26^Mg* and μ^54^Cr signatures of CM chondrites are clustered together with compositions typical of their own group, the anomalous CM chondrite Bells is more similar to CR chondrites, with typically negative μ^26^Mg* and positive μ^54^Cr signatures (Fig. 1A).

### CM Chondrules

**Figure 3:**
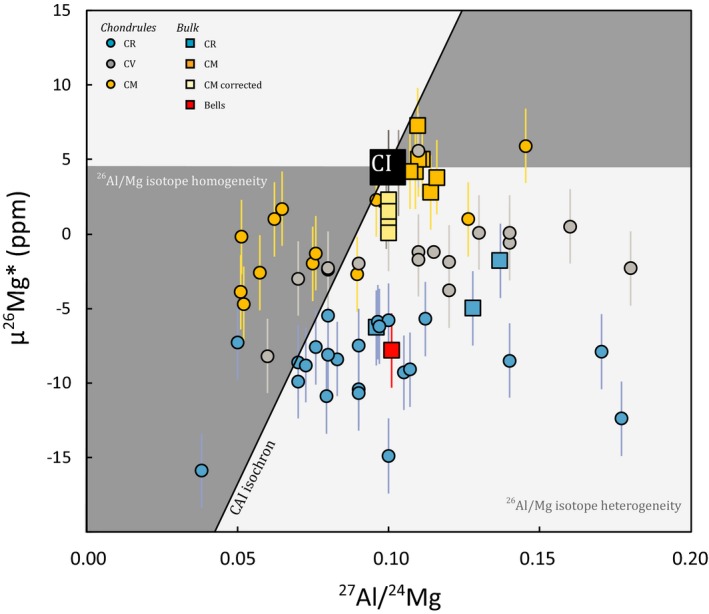
Plot modified after Olsen et al. (2016; their fig. 5) showing ^27^Al/^24^Mg versus μ^26^Mg* for CR; CV (Olsen et al. 2016), and CM chondrules (this work, Table 1); and bulk CR, CI, and CM chondrites including Bells (Van Kooten et al. 2016, this work). The dark gray fields mark the data where ^26^Al and/or Mg isotope homogeneity are allowed. These fields are barred by the CAI‐AOA isochron identified by Larsen et al. (2011) and by a horizontal line where all ^26^Al has decayed. Error bars reflect the external reproducibility of 2.5 ppm and 2% for the μ^26^Mg* and ^27^Al/^24^Mg values, respectively. (Color figure can be viewed at http://www.wileyonlinelibrary.com.)

We have determined the Mg and Cr isotope composition of 12 chondrules from the CM chondrite Jbilet Winselwan. The μ^26^Mg* values range between −4.7 ± 2.1 ppm and 5.9 ± 2.0 ppm (Table 1), whereas the μ^54^Cr compositions have values between −11 ± 20 and 130 ± 40 ppm. These chondrule compositions are plotted in Fig. 1B, together with the CV and CR chondrule Mg and Cr isotope signatures as well as compositions of bulk chondrites from Fig. 1A. This plot suggests that the Mg and Cr isotope signatures of CM chondrules overlap with those of CV chondrules, whereas CR chondrules seem to be distinctly different. In addition, histograms of μ^26^Mg* and μ^54^Cr values for CR, CV, and CM chondrules show that the chondrule frequency distributions are similar for CV and CM chondrules but distinct for CR chondrules. To quantify these observations, we have calculated the statistical significance of different chondrule populations using a t‐test (if the chondrule populations are normally distributed) and a Mann–Whitney U‐test (if the distribution is assumption‐free). The null hypothesis in these tests reflects chondrule subsamples being drawn from the same population. We show that in both tests, the null hypothesis can be accepted for CM and CV chondrule populations, but is rejected for CM and CR chondrule populations (Table 2). In other words, the results confirm that CV and CM chondrules have identical populations in μ^26^Mg* and μ^54^Cr space, but that CM and CR chondrule populations are significantly variable. We further show in Fig. 3A that variations in μ^26^Mg* between CV/CM and CR chondrules are not related to differences between their ^27^Al/^24^Mg ratios. CM chondrules measured in this study mostly have low, subsolar ^27^Al/^24^Mg ratios ranging between 0.051 and 0.145 with an average of 0.079 ± 0.061. CR chondrules with a similar range of ^27^Al/^24^Mg ratios have typically lower μ^26^Mg* values. Last, we have measured the ^55^Mn/^52^Cr ratios and μ^53^Cr values of 10 CM chondrules. The chondrules typically have lower ^55^Mn/^52^Cr ratios, ranging between 0.128 and 0.356, as well as lower μ^53^Cr values, ranging between −9 ± 6 ppm and 22 ± 4 ppm, relative to bulk CM chondrites (Table 1).

**Table 2:** Results from testing the significance of variations between μ^26^Mg* and μ^54^Cr values of CR, CV, and CM chondrule populations. Statistical calculations are done using the Mann–Whitney U‐test and the t‐test

	μ^26^Mg*		μ^54^Cr	
	CV‐CM	CR‐CM	CV‐CM	CR‐CM
Mann–Whitney U‐test U	98.5	0	95.5	3
Z	−0.61	−4.78	0	4.53
*p*	0.54	*<*0.00001	1	*<*0.00001
T‐test t	0.87	8.43	0.084	−8.9
*p*		*<*0.00001		*<*0.00001
Results (significantly different)	No	Yes	No	Yes

## Discussion

### The Significance of Mg and Cr Isotope Variability Between Bells and Other CM Chondrites

The μ^54^Cr variations in bulk solar system materials have been interpreted as reflecting accretion of these materials in isotopically different reservoirs of the proto‐planetary disk (Trinquier et al. 2007, 2009; Qin et al. 2010; Warren 2011). Generally, increasing μ^54^Cr values are correlated with a larger orbital distance, the main reason being that carbonaceous chondrites, which are thought to have formed in the outer solar system, are enriched in ^54^Cr relative to noncarbonaceous chondrites. Moreover, CI chondrites, which are considered the least thermally processed meteorites with solar chemical composition, have the highest μ^54^Cr values. In this model, CR chondrites are considered intermediate compositions between pristine CI chondrites and more thermally processed solar system materials and should thus have accreted within the orbit of the CI chondrite parent body. However, recent analyses of μ^26^Mg* values from bulk CR chondrites and CR chondrules (and other MRCCs including CH, CB chondrites and chondritic lithic clasts from Isheyevo) show a distinct decoupling between μ^26^Mg* and μ^54^Cr values of CR chondrites and other solar system materials (Van Kooten et al. 2016). Since the μ^26^Mg* value is mostly dependent on the decay of ^26^Al and, consequently, the Al/Mg ratio, some variation could potentially be caused by Al/Mg fractionation or CAI incorporation. However, CR bulk chondrites and chondrules along a range of Al/Mg ratios, including a solar CI‐chondrite ratio, are found to have a similar offset in μ^26^Mg* relative to CI, CM, and CV chondrites (Fig. 3A). This implies that the lower μ^26^Mg* values of MRCCs are the result of either ^26^Al or Mg isotope heterogeneity and that Bells, having a similar composition, also accreted material that is isotopically distinct from other chondrites and solar system objects (Fig. 1A). As outlined in previous work (Larsen et al. 2011, 2016a; Schiller et al. 2015a; Olsen et al. 2016; Van Kooten et al. 2016; Bollard et al. 2017; Connelly et al. 2017), μ^26^Mg* variations in bulk solar system materials and chondrules have been interpreted to reflect ^26^Al heterogeneity. However, whether the observed μ^26^Mg* variability reflects ^26^Al or Mg‐isotope heterogeneity is not relevant for our interpretations in terms of accretion region: MRCCs still must have accreted in an isotopically distinct reservoir in the protoplanetary disk. As such, we interpret our CM data in the framework of the accretion model proposed by Van Kooten et al. (2016). In ^26^Mg*‐^54^Cr space, the anomalous Bells CM chondrite plots, together with MRCCs, on the array extending from the solar system's ^26^Mg*‐^54^Cr correlation line toward the ^26^Mg*‐depleted and ^54^Cr‐enriched component inferred to represent thermally unprocessed primordial molecular cloud material. At face value, this observation suggests that Bells incorporated significant amounts of thermally unprocessed primordial molecular cloud material, unpolluted by ^26^Al. This is in sharp contrast with other bulk CM chondrites, as well as CI and CV chondrites with similar μ^26^Mg* values (Larsen et al. 2011; Chen et al. 2018).

### Evidence for Protracted Accretion in the CM Chondrite Reservoir

**Figure 4:**
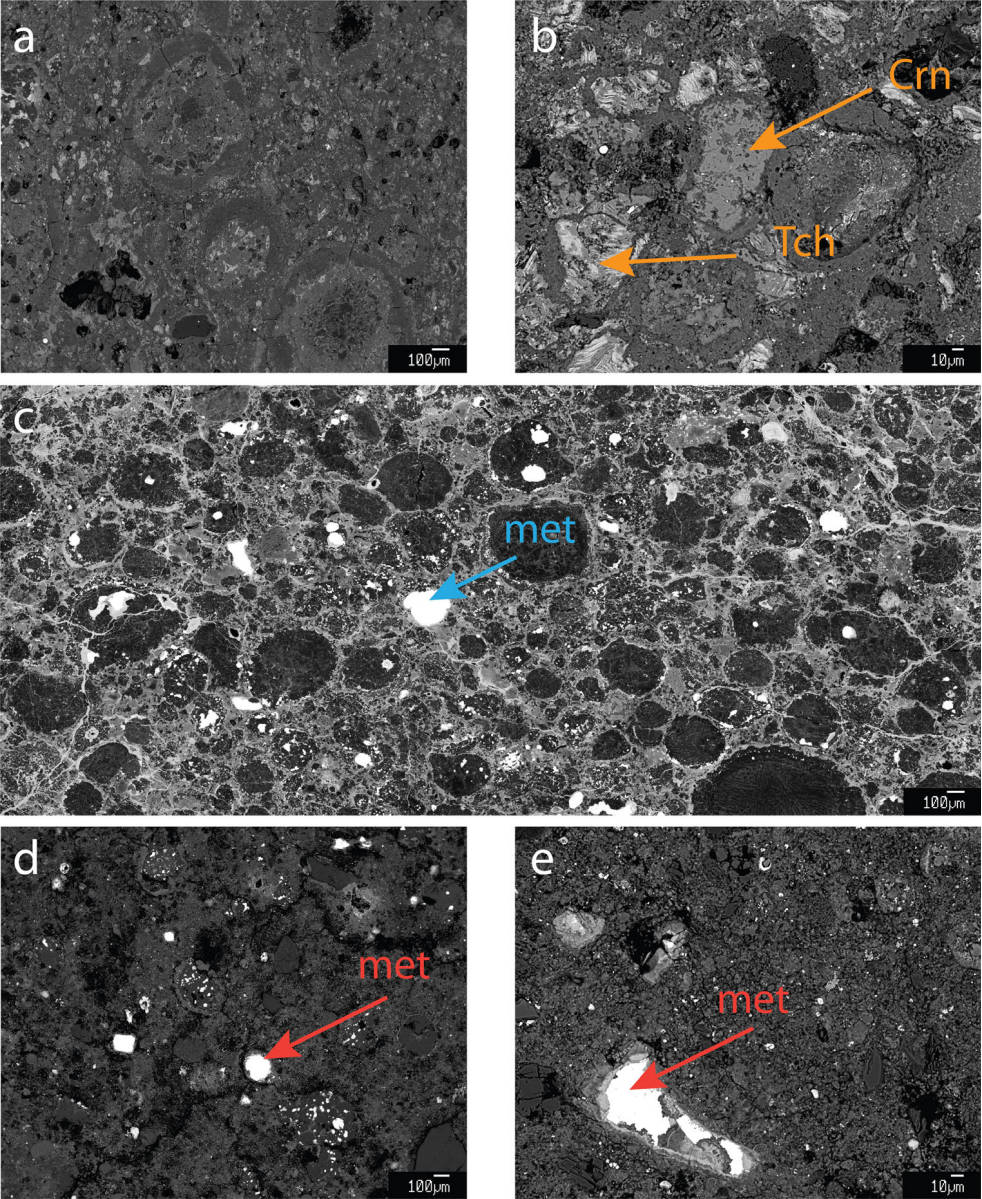
Backscattered electron images of (a) the moderately altered CM chondrite Jbilet Winselwan showing rimmed chondrules in a tochilinite‐cronstedtite‐intergrowth‐rich matrix; (b) the relatively unaltered CM chondrite Maribo, also with abundant tochilinite (Tch) and cronstedtite (Crn); (c) CR chondrite NWA 6043 with abundant FeNi metal (met); (d‐e) CM chondrite Bells also contains abundant metal grains (for additional images, see also Van Kooten et al. 2018). Metal in altered CM chondrites is usually not present (Rubin et al. 2007) and the most unaltered CM chondrites Paris and Maribo have metal abundances of ~3 vol% (Hewins et al. 2014; Van Kooten et al. 2018). (Color figure can be viewed at http://www.wileyonlinelibrary.com.)

The ^26^Mg* and ^54^Cr isotope compositions of Bells suggest a genetic relationship to MRCCs. Furthermore, Bells’ insoluble organic matter (IOM) records high ^15^N/^14^N values (Alexander et al. 2007; Van Kooten et al. 2018) and its mineralogy, even though heavily altered, includes a significant amount of FeNi metal (Figs. 4c–4e), which are features typically observed in CR chondrites. This high abundance of metal and the high degree of aqueous alteration observed in Bells may also explain its high abundance of magnetite (11–13 vol%; Watson et al. 1975; Hyman and Rowe 1983; Davis and Olsen 1984) relative to typical CM chondrites (1–2 wt%; Hyman and Rowe 1983; Howard et al. 2011). Moreover, the high degree of aqueous alteration could have led to the decomposition of tochilinite–cronstedtite intergrowths distinctive of CM chondrites (Figs. 4a and 4b), which would have reacted to form serpentine and magnetite (Bach et al. 2006; King et al. 2017; Van Kooten et al. 2018). Regardless of these close affinities to CR chondrites, Bells is classified as a CM chondrite, albeit an anomalous one (Mittlefehldt 2002), since it shares with other CM chondrites a common oxygen isotope composition of magnetite and matrix minerals (Rowe et al. 1994), similar noble gas signatures (Zadnik 1985), a similar bulk chemical composition (apart from a depletion of refractory elements due to a lack of CAIs, Brearley 1995; Mittlefehldt 2002), and a common hydrogen isotope composition of matrix phyllosilicates (Van Kooten et al. 2018). These characteristics suggest that Bells and other CM chondrites were aqueously altered under similar conditions and fluid composition, which indicates that Bells and other CM were either derived from the same parent asteroid or at least from CM‐like asteroids that accreted within the orbital vicinity of the CM chondrite parent body (Lee et al. 2019). It is highly unlikely that such a similarity in chemical and isotopic features can be recreated in a different chondritic reservoir (e.g., at significantly different orbital distances or time), considering the already large variations found within CCs, let alone within different chondrite groups.

**Figure 5:**
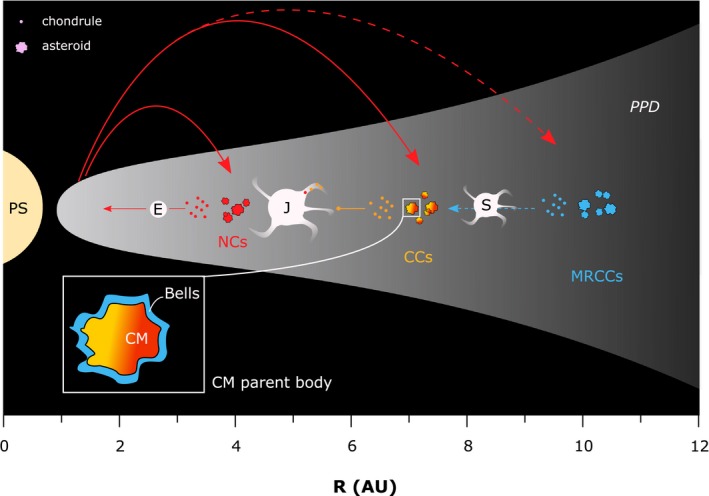
Schematic overview of the protoplanetary disk with its protosun where planet formation occurs and *E* is Earth, *J* is Jupiter with accretionary filaments, and *S* is Saturn. Transport of chondrules is represented by arrows. The dashed blue arrow reflects transport through a semipermeable barrier, whereas the orange arrow reflects a nonpermeable barrier. The red arrows represent chondrule transport from the inner solar system. (Color figure can be viewed at http://www.wileyonlinelibrary.com.)

The Mg and Cr isotope signatures of Bells imply that this meteorite incorporated the same ^26^Al/^26^Mg*‐poor, ^54^Cr‐rich component as CR chondrites, in addition to accreting similar IOM and abundances of metal. This apparent paradox can be resolved by invoking protracted accretion in the CM chondrite reservoir, during which thermally unprocessed primordial molecular cloud matter originating from the outer solar system is progressively accreted to the CM parent body or CM‐like asteroids over time. This protracted accretion is in agreement with the pebble accretion model and supporting cosmochemical data in which pebble‐sized objects continuously accrete to asteroids assisted by gas drag during the lifetime of the protoplanetary disk (Johansen et al. 2015; Larsen et al. 2016b). Hence, we suggest that the anomalous CM chondrite Bells represents a relatively late‐accreted outer layer of chondritic material to the CM parent body or CM‐like asteroids (Fig. 5). We reason that within a model of heterogeneously distributed ^26^Al, this ^26^Al‐poor (^26^Al/^27^Al_0_ = 1.05 × 10^−5^, Van Kooten et al. 2016) MRCC‐like material must have been added late rather than early to the typical, relatively ^26^Al‐rich (^26^Al/^27^Al_0_ = 2.83 × 10^−5^, Larsen et al. 2011) composition of the CM chondrite reservoir. This is supported by Mo isotope analyses of iron meteorites, which suggest that some groups of iron meteorites represent the once molten cores of carbonaceous chondrite parent bodies (Burkhardt et al. 2011). If these planetesimals would have had an ^26^Al/^27^Al_0_ akin to the MRCC reservoir and Bells‐like CM material, it is highly unlikely that these bodies would have melted and differentiated. Alternatively, if the ^26^Mg* variations between Bells and other CM chondrites reflect Mg isotope instead of ^26^Al isotope heterogeneity, we still argue for a late addition of Bells to CM or CM‐like chondrite parent bodies. In this scenario, a homogeneous distribution of ^26^Al in the protoplanetary disk reflects the relatively late accretion of CR chondrites (Schrader et al. 2017; Budde et al. 2018). Consequently, the fact that Bells has the same Mg and Cr isotope composition as CR chondrites and other metal‐rich carbonaceous chondrites implies that Bells also accreted late. Hence, the most probable scenario is that Bells represents a late addition to the CM chondrite parent body or, alternatively, to an asteroid accreted within a family of CM‐like asteroids.

Such a scenario is compatible with the O and H isotope composition of Bells, since these signatures are largely controlled by the composition of ices (i.e., water and ammonia) for which the stability depends on radial distance in the protoplanetary disk. For example, CM chondrites, including Bells, are inferred to have accreted a lesser amount of NH_3_ ice (Monroe and Pizzarello 2011; Van Kooten et al. 2018) than MRCCs (i.e., CR chondrites: Pizzarello et al. 2008; Pizzarello and Yarnes 2016 and Isheyevo chondritic clasts: Van Kooten et al. 2017a), suggesting that the CM parent body, including Bells, accreted sunward of the NH_3_ ice line. This implies that the timescale of accretion of the CM parent body and Bells was protracted and limited to the timescale of inward movement of the NH_3_ ice line, which would have moved sunward toward the asteroid belt within 2 Myr (Dodson‐Robinson et al. 2009). This provides an upper limit for the accretion timescale of the CM parent body or asteroids in the CM chondrite reservoir. We note that in the ice line model of Dodson‐Robinson et al. (2009), NH_3_ and H_2_O ice lines are located at the same orbital distance, since they have been assigned similar binding energies. However, the binding energies of NH_3_ and H_2_O are linked to the material on which they condense. For example, the binding energy of NH_3_ on H_2_O ice is higher than for NH_3_ on NH_3_ ice. Hence, a mixture of NH_3_ and H_2_O ice would lead to a separation of the two ice lines at the disk midplane, with the NH_3_ ice line being located at larger orbital distances relative to the H_2_O ice line (Ecrenaz 2008; Eistrup et al. 2016). The limit provided by the NH_3_ ice line migration is in agreement with ^53^Mn‐^53^Cr ages from secondary calcite and dolomite in CM chondrites (Fujiya et al. 2012; Jilly et al. 2014), which suggest that aqueous alteration occurred on average around 3–4 Myr after CAI formation and perhaps as early as >2.4 Ma after CAI formation based on uncertainties within the Sutter's Mill isochron (Jilly et al. 2014) and consequently placing the accretion of the parent body before this time. Thermal modeling of the CM chondrite parent body invokes heating by decay of ^26^Al, which assumes a canonical ^26^Al/^27^Al ratio, resulting in an accretion age of 3.0–3.5 Myr after CAI formation (Fujiya et al. 2012). We note that using a reduced ^26^Al/^27^Al abundance in the CM chondrite accretion region, as inferred by Larsen et al. (2011), would place the accretion of this body at earlier times, in close agreement with the timing of ice line migration (Dodson‐Robinson et al. 2009).

Chondrule formation ages reflect a lower limit for the accretion timescale of chondrites. Chondrules from CM chondrites have not been dated, since these chondrules are generally too small (~270 μm, Friedrich et al. 2015) for U‐isotope corrected Pb‐Pb dating. Furthermore, even assuming a homogeneous ^26^Al/^27^Al ratio in the protoplanetary disk, CM chondrules (probably with the exception of Paris and Maribo chondrules) are too altered to infer meaningful Al‐Mg ages. However, it may be possible to use CV chondrules as a proxy for CM chondrule ages. Based on similarities in Mg and Cr isotope ranges, we expect chondrules from CV chondrites to have formed in the same region as chondrules from CM chondrites (Fig. 2). So far, limited assumption‐free age information is available for CV chondrules. Only two individual chondrules from the CV chondrite Allende have been Pb‐Pb dated so far, with ages of 4567.32 ± 0.42 Ma and 4566.24 ± 0.63 Ma (Connelly et al. 2012; Bollard et al. 2017), as well as a multi‐chondrule fraction from Allende with a U‐isotope corrected age of 4564.32 ± 0.81 Ma (Connelly and Bizzarro 2009). A recent study using the Al‐Mg dating system and assuming a homogeneous distribution of ^26^Al in the solar system suggests that CV chondrules formed as early as *<*0.5 Ma after CAI formation (Chen et al. 2018). Using a reduced abundance of ^26^Al in the CV chondrule region would only limit chondrule formation to older ages, in agreement with individual Pb‐Pb ages of CV chondrules. We note that average CV chondrule ages using the Hf‐W decay system (~2.2 Myr after CAI formation, Budde et al. 2016) do not provide a lower limit for the accretion age and have recently been refuted. Connelly et al. (2017) show that the ^182^W versus Hf/W correlation of CV chondrite components is a mixing line and does not have chronological significance. Collectively, these data suggest that CV chondrule formation occurred at least until 3 Myr after CAI formation. However, the statistics of Pb‐Pb dated individual and relatively unaltered CV chondrules is nonexistent at this time and additional data are needed to constrain the lower limit of accretion timescales for the CV and by proxy, the CM chondrite parent body. It is, for example, unclear how the relatively high degree of secondary alteration within Allende could have affected the U‐Pb isotope systematics. In addition, most CV chondrules may have formed rapidly after CAI formation, whereas only some outliers formed at later times, similar to chondrules from ordinary chondrites (Bollard et al. 2017). These statistical limitations show that the secondary alteration ages of CM chondrites define more robust constraints for the accretion timescales. Hence, we propose that the CM chondrite parent body accreted <3 Myr after CAI formation and—taking into account the timescales of ice line migration and a reduced initial abundance of ^26^Al/^27^Al—possibly as early as <2 Myr after CAI formation. Consequently, an outer layer of Bells‐like material on the CM body or CM‐like bodies including ^26^Al/^26^Mg*‐poor primordial molecular cloud matter, suggests that this matter was mixed into the disk around 2–3 Myr.

### Reservoirs of CM, CV, and CR Chondrule Populations

Earlier, we have mentioned the use of CV chondrules as a proxy for CM chondrules. Based on their similarities in Mg and Cr isotope compositions, we suggest they must have occupied a similar accretion region. The range of μ^26^Mg* and μ^54^Cr values implies that some chondrules were derived from the inner solar system (low μ^54^Cr values) and transported outward to the accretion region of carbonaceous chondrites, whereas other chondrules were derived from the outer solar system (high μ^54^Cr values, Olsen et al. 2016). Recently, Gerber et al. (2018), following Trinquier et al. (2009), have attributed Ti isotope variations in CV chondrules to incorporation of CAI‐like materials. Similar to Ti isotope variations in chondrules, they suggest that other nucleosynthetic isotope variations, including ^54^Cr, are nothing more than nugget effects. They propose that chondrule precursor dust was at some point variably enriched in “nuggets” of ^54^Cr‐rich dust, after which chondrule formation took place. As a consequence, Ti and Cr isotope variations in chondrules reflect only the variability of the precursor dust in the chondrule forming reservoir. This is opposed to the model where transport of chondrules with variable μ^54^Cr values to their final accretion regions leads to ^54^Cr variability of individual chondrules within the same chondrite (Olsen et al. 2016). The model by Gerber et al. (2018) would nullify our proposition to use CV chondrules as a proxy for CM chondrules. However, besides the lack of empirical evidence, the model by Gerber et al. (2018) predicts that the ^54^Cr variations in mm‐sized chondrules should be larger than differences between bulk solar system materials such as chondrites and achondrites, which represent km‐sized planetary bodies. On the contrary, Bizzarro et al. (2017) show that the μ^54^Cr variation between chondrules is of the same scale as that of bulk chondrites and achondrites (see their figure 6.10), whereas the size of chondrules is three orders of magnitude smaller than that of asteroids, the parent bodies of chondrites. This observation establishes that the ^54^Cr variability in chondrules cannot reflect a nugget effect related to the incorporation of an unidentified anomalous presolar carrier. Hence, we suggest that the μ^54^Cr and μ^26^Mg* CV and CM chondrule data reflect accretion of chondrules to CV and CM chondrites, sampled from the same reservoir. Although the physical properties of CV and CM chondrules are different (e.g., size, compound chondrules, igneous rims, abundance of RP and C chondrules; Rubin 2010), the overall petrology, Cr, Mg, and O isotope composition, and Mg number of these chondrule populations (this work, Chaumard et al. 2018) suggest that they were likely derived from similar chondrule forming regions in the protoplanetary disk. Perhaps, within these regions, different local environments existed with varying conditions or chondrule forming mechanisms. Furthermore, chondrules could already have been size sorted and stored beforehand in different chemical reservoirs and subsequently been transported to their accretion regions. Alternatively, the fact that CM chondrules (~270 μm, Friedrich et al. 2015) are typically smaller than CV chondrules (~910 μm, Friedrich et al. 2015) may reflect a size sorting during accretion of CV and CM chondrite parent bodies (Johansen et al. 2015).

We have further shown that CR chondrules represent a significantly different population relative to CM and CV chondrules, having lower μ^26^Mg* and higher μ^54^Cr values (Fig. 2). Moreover, we have demonstrated that the lower μ^26^Mg* values of CR chondrules must be the result of ^26^Al or Mg isotope heterogeneities between CR and CM/CV chondrule forming reservoirs. Since we argue that Bells, with a similar isotopic signature, represents a relatively late accreted layer of the CM chondrite parent body, it would imply that CR chondrules have formed later than CM/CV chondrules. This seems to be in agreement with young average Al‐Mg and average Pb‐Pb CR chondrule ages between 3.5 and 4 Ma (Amelin et al. 2002; Schrader et al. 2013, 2017; Nagashima et al. 2014), relative to CV chondrules, which may on average be older (Amelin and Krot 2007; Connelly and Bizzarro 2009; Luu et al. 2015; Chen et al. 2018). Nevertheless, while it may be that CR chondrules as a population formed later than CV chondrules, assumption‐free U‐isotope corrected Pb‐Pb ages of individual chondrules show that a fraction of these chondrules formed simultaneously or rapidly after CAI formation (Bollard et al. 2017). Since CV and CR chondrules consist of distinct populations in μ^26^Mg* and μ^54^Cr space, this suggests that the chondrule‐forming reservoirs were initially spatially separated. For example, if CR and CV chondrules formed in the same reservoir, but at different times, CR chondrites would be expected to (1) have only young chondrules with relatively high μ^54^Cr values or (2) also have old chondrules, a fraction of them being relatively ^54^Cr‐poor. Since neither is true, the CR chondrule reservoir must have been spatially separated from that of CV and CM chondrules. We note that this does not exclude late accretion of CR chondrites relative to CV and CM chondrites.

### A Hypothesis on the Nature of Barriers Between Chondrite Accretion Regions

The ^26^Mg*‐^54^Cr systematics of individual chondrules from CM, CV, CR, CH, and CB chondrites (Olsen et al. 2013, 2016; Van Kooten et al. 2016) suggest that chondrules from MRCCs and CV/CM chondrites formed in distinct reservoirs. Importantly, some CV and CR chondrules have Pb‐Pb isotope ages that reflect early formation simultaneously with CAIs (Bollard et al. 2017). Collectively, this suggests that initially the formation of MRCC chondrules occurred in a reservoir spatially isolated from CV chondrites and by extension CM chondrites (Fig. 5). Although evidence points toward a late accretion of MRCCs relative to CM and CV chondrites in this study and in previous work (Bollard et al. 2015; Budde et al. 2016, 2018; Schrader et al. 2017; Van Kooten et al. 2017a, 2019), it is also apparent that CR and CV/CM chondrule formation preceding chondrite accretion occurred in distinct reservoirs. Hence, the presence of “old” chondrules in CR chondrites necessitates the existence of a physical barrier isolating the two reservoirs. However, around 2–3 Myr after CAI formation, ^26^Al/^26^Mg*—poor material was mixed into the CM chondrite reservoir, as evidenced by the composition of the anomalous CM chondrite Bells. This implies that the barrier would have initially separated two chondrule‐forming reservoirs, but would at some point become more permeable, allowing for the inward transfer of submicron‐sized organic matter as well as mm‐sized chondrules or their precursor dust. This barrier would not have allowed for significant mass transport during the accretion of CCs, but would after *<*3 Myr become more permeable to facilitate the inward transport of organic grains and ^26^Al/^26^Mg*‐poor dust and/or chondrules. In the following paragraph, we speculate about the nature of this barrier and its possible implications for the dynamics of the protoplanetary disk.

The accretion of planets and the resulting regions in the protoplanetary disk depleted of gas and dust (i.e., planetary gaps) are obvious barriers to separate dust reservoirs. For example, the formation of Jupiter has been put forward to explain isotope heterogeneities between CCs and other solar system objects (Budde et al. 2016; Kruijer et al. 2017). Similarly, as considered below, other gas giants such as Saturn may pose as a barrier between CCs and MRCCs. Intuitively, such a barrier is expected to become less permeable over time, as the gas is depleted and the gap grows. Whilst the transition of dust through such a gap is influenced by many factors (e.g., grain size, turbulent viscosity, planet mass, stellar accretion rate; Paardekooper and Mellema 2006; Rice et al. 2006; Pinilla et al. 2012), dust filtration is found to be more efficient when the gap is deep and narrow, resulting in a higher pressure gradient at the outer edge of the gap (Weber et al. 2018). In general, the more massive a planet is, the smaller the particles that can reach the inner edge of the gap (Weber et al. 2018). Hence, a growing planet is expected to filter more particles over time, a trend opposite to expectations brought forward by our data. Furthermore, this filter becomes more effective when the stellar accretion rate and surface density of the gas decrease during the aging of the disk (Weber et al. 2018). However, as we discuss below, the profile of a planetary gap can be affected in multiple ways to accommodate changes in dust filtration efficiency.

We first consider the effect of turbulent viscosity on the density profile of a planetary gap. With increasing viscosity in the disk, more gas is able to refill the planet‐carved gap, and consequently, the steepness of the density profile becomes less pronounced, allowing for more efficient dust transitions (Weber et al. 2018). Similarly, it is found that high‐viscosity disks have a higher pebble isolation mass (PIM: the mass needed for a planet to exclude pebbles from the gap area, Ataiee et al. 2018). A small PIM implies that it only requires a small planetary mass to perturb the disk sufficiently to expel pebbles from the planet's accretion area. A large PIM means that a higher planetary mass is necessary to have the same effect on the disk and for the planet to serve as an efficient barrier. In terms of turbulent viscosity, this implies that with a constant planet mass, an increasingly viscous disk would reduce the efficiency of dust filtration over time. Turbulence can result from magnetorotational instabilities in the disk and acts as a mechanism to transport angular momentum outward. This turbulence is expected to increase over the lifetime of the protoplanetary disk since, with time, non‐thermal ionization (e.g., X‐rays from the protosun and interstellar cosmic rays) becomes more efficient through dissipation of the gas (Turner and Drake 2009). A planet with a Saturn mass would initially be large enough to filtrate mm‐sized particles, but would over time become a less efficient barrier due to increasing turbulence (Weber et al. 2018). Jupiter would be massive enough not to be affected in the mm‐sized particle regime. Hence, the accretion of Saturn could act as an effective semipermeable barrier between CCs and MRCCs. Planet migration may affect the permeability of the gap by changes in relative velocity between migration rate and the viscous flow of the gas (Weber et al. 2018). In other words, if Saturn initially migrates in the type II regime, its radial velocity is connected to the viscous flow of the gas. This would reduce the relative velocities with which the well‐coupled dust particles approach the gap, limiting their transport inside of Saturn's orbit. If at some point Saturn's migration stopped, or even turned outward, the efficiency of dust filtration by the planetary gap would accordingly become weaker, allowing also larger sized grains to be transported toward the inner regions. Although this relationship between migration and gap permeability is subject to further study, this may be interesting in terms of timing of planetary migration models such as the Grand Tack (Walsh et al. 2011). Headway has been made to date the onset of Jupiter's accretion (i.e., *<*1 Ma, Van Kooten et al. 2016; Kruijer et al. 2017), but so far cosmochemical data as a tool to assess the timing of the potential migration of the gas giants as put forward by astrophysical models (Walsh et al. 2011) is limited. It has been suggested that Jupiter's migration occurred around 5 Ma (Johnson et al. 2016), in order to explain the CB chondrites that are products of high‐velocity impact gas/melt plumes (Krot et al. 2005). We suggest that, if the Grand Tack model is correct and the cause of Saturn's decreasing dust filtration, our data hint toward the onset of outward gas giant migration at 2–3 Ma. Finally, we note that the investigation of other anomalous chondrites such as Bells may strengthen our results and work toward the verification of this hypothesis.

## Conclusions

We report on the Mg and Cr isotope systematics of seven bulk CM chondrites and 12 CM chondrules. The main conclusions of this work can be summarized as follows:

1. We show that Bells, although sharing many chemical characteristics with CM chondrites, has a Mg and Cr isotope signature similar to MRCCs. This implies that Bells accreted on the CM chondrite parent body, albeit at a later time. Based on the timing of chondrule formation, aqueous alteration of the CM chondrite parent body, and the limited presence of NH_3_ ice relative to MRCCs, we propose protracted accretion of the CM chondrite parent body, during which ^26^Al/^26^Mg*‐poor dust from the MRCC reservoir was added to the CM accretion region between 2 and 3 Myr after CAI formation.
2. Our results further show that in Mg and Cr isotope space, CM and CV chondrule populations are indistinct from one another, whereas CR chondrules have significantly different signatures. We therefore suggest that CM and CV chondrules formed in a spatially separated reservoir from CR chondrules.
3. While Mg and Cr isotope data point toward late accretion of CR relative to CM/CV chondrites, individual Pb‐Pb ages of CR and CV chondrules show that initially CM and CV chondrules formed in a spatially separated reservoir from CR chondrules. We therefore hypothesize on the nature of the barrier separating the two reservoirs and speculate that the barrier isolating the MRCC from the CC reservoir was formed by the accretion of Saturn. While initially Saturn would act as an effective barrier for submillimeter‐sized particles, outward migration would increase the permeability of the planetary gap, allowing for a flux of ^26^Al/^26^Mg*‐poor dust toward the inner solar system. We expect this next generation of dust to be visible in other ungrouped and anomalous chondrites than Bells.

## Editorial Handling

Dr. Gretchen Benedix
